# Measuring recovery in high-security patients: psychometric evaluation of the Questionnaire about the Process of Recovery and its utility to assess the forensic recovery journey

**DOI:** 10.1192/bjo.2025.10941

**Published:** 2026-01-22

**Authors:** Lindsey Gilling, Cheryl Rees, Lindsay D. G. Thomson

**Affiliations:** Division of Psychiatry, University of Edinburgh, Edinburgh, UK; Medical Department, The State Hospitals Board for Scotland, Carstairs, UK

**Keywords:** Forensic psychiatry, mental health services, clinical outcomes measures, recovery, psychometrics

## Abstract

**Background:**

Forensic mental health services need a reliable and repeatable outcome measure to assess the progression of self-rated recovery during the forensic journey. The Questionnaire about the Process of Recovery (QPR) was developed in individuals with psychosis, and has been used to assess recovery in people with severe mental illness; however, its psychometric properties have not been studied in a forensic psychiatric cohort.

**Aims:**

This study aimed to assess the psychometric properties of the QPR in a sample of individuals who currently access, or formerly accessed, high-security psychiatric care, including internal consistency, test–retest reliability, factor structure and criterion validity.

**Method:**

Psychometric analysis was undertaken in a sample of 146 current or former high-security patients. Confirmatory and exploratory factor analysis examined the latent test structure. Non-parametric comparisons of QPR score indices tested for differences according to individuals’ current setting (high-, medium- or low-security or open wards; community) as evidence of criterion validity.

**Results:**

A unique two-factor structure related to self-actualisation/empowerment and growth/insight fit forensic patients’ QPR responses. Internal consistency and test–retest reliability were adequate for QPR all-item scores for the original and shortened scales, as well as for the new forensic factor scores. QPR score indices differentiated patients by current setting (eta^2^ = 0.03–0.04), although only the forensic factor related to growth/insight was significant in corrected *post hoc* comparisons.

**Conclusions:**

The original QPR is recommended for use to assess recovery progress in a forensic psychiatric sample. Forensic patients’ scores may be best represented using the unique two-factor structure identified.

Individuals are detained within forensic mental health services due to their violent or criminal propensities and experience of severe mental disorder. They form an often-marginalised population who can experience stigma, both self and societal, in relation to their mental disorder and offending behaviour.^1^ These individuals’ backgrounds are often laden with adverse events and disadvantage.^2^ Given this, forensic mental health services have increasingly embraced person-centred practices that aim to provide individual care and treatment plans that foster hope, control and opportunity as the central tenets of recovery.^3^ An individual’s recovery journey is a deeply personal process that requires the person to take ownership and actively engage with the recovery process.^4^

There is a need within forensic mental health services for reliable, easily delivered outcome tools^5–7^ that can be repeated to measure and explore the progression of self-rated recovery. Subjective assessment of recovery is essential to inform services as how best to support individuals and further develop recovery-orientated services.^8^ While there is a diversity of recovery measures available, few have had their psychometric properties robustly assessed^9,10^ and no published measures have been specifically designed to examine recovery among individuals cared for within forensic mental health services. It may seem intuitive to develop an outcome measure specifically tailored to the forensic population, but as a first step it is proposed to evaluate a psychometrically robust measure already widely used across culturally and linguistically diverse groups. The Questionnaire about the Process of Recovery (QPR)^11^ was developed within the UK, and was informed by service users with experience of psychosis.^12^ This is an important consideration, because in Scotland 64% of forensic mental health service patients are diagnosed with a schizophrenia.^13^ In addition, the psychometric properties have been extensively examined demonstrating, as a minimum, good internal consistency, construct validity and test–retest reliability.^8,11,14^ The QPR is recommended for use by the UK National Institute for Health and Care Excellence,^15^ and has been assessed in diverse groups^16^ and developed internationally.^17–21^ To evaluate the usefulness of the QPR to assess recovery within a forensic population, this study tested for differences in self-rated recovery scores among individuals currently accessing high-security care, and among those who are former high-security patients and have stepped down to less secure settings, including in the community. It also explored the measure’s internal reliability and test structure for the first time within a forensic population.

## Method

### Procedure

Anonymised QPR data and limited patient information, originally collected in three studies related to forensic recovery, were collated and analysed in the current study (*N* = 146). Two-week test–retest reliability was assessed in a sample of 50 high-security patients recruited for this purpose. Additional detail regarding the origin of these data and administration of the QPR in the original studies is available in the supplementary material available at https://doi.org/10.1192/bjo.2025.10941.

The authors assert that all procedures contributing to this work comply with the ethical standards of the relevant national and institutional committees on human experimentation, and with the Helsinki Declaration of 1975 as revised in 2013. The South East Scotland Research Ethics Service confirmed National Health Service (NHS) ethical review was not required because the present study was limited to the secondary use of anonymised data. The State Hospital Research Committee approved the study, and hospital managerial approval was received for the study to commence. In the original collection of these data-sets, written participant consent to participate in research was obtained (studies 1 and 2) and, in study 3, written consent was not sought because the data were collected in the course of routine clinical care, although the State Hospital patient privacy notice covered secondary use of these data.

### Sample characteristics

All participants were current or former patients of the State Hospital in Carstairs, the high-security hospital for Scotland and Northern Ireland. All participants were male. Nearly three-quarters of participants (74.7%, *n* = 109) were currently in high-security care; 18 (12.3%) had stepped down from high security and were receiving in-patient care in general or forensic wards in medium or low security; and 18 (12.3%) had been discharged from in-patient services and were residing in the community at the time of assessment. Sample characteristics data are available for *n* = 145, and summary characteristics of the sample are presented in Table 1. Participants differed in age (*χ* ^2^(2) = 43.84, *P* = <0.001) and years spent in their current setting (*χ* ^2^(2) = 19.13, *P* < 0.001), according to their current setting. A majority of the sample (78%, *n* = 114) were diagnosed with a psychotic disorder. The most common secondary diagnoses were personality disorders (22.6%) and substance use disorders (21.9%).

**Table 1:** Sample characteristics

Variable	High	Medium-/low-security/open	Community	All^a^
*n*	109	18	18	145
Age (median, IQR)	38.00 (15.00)	54.00 (9.00)	55.50 (12.00)	43.00 (20.00)
Years in current setting (median, IQR)	1.69 (3.20)	3.17 (7.45)	8.04 (11.61)	2.17 (4.67)
Primary diagnosis (*n*, %)				
Psychotic disorder	94 (86.20)	8 (44.40)	12 (66.67)	114 (78.10)
Affective disorder	6 (5.50)	--	--	6 (4.10)
Anxiety disorder	1 (0.92)	--	1 (5.60)	2 (1.40)
Personality disorder	2 (1.83)	8 (44.44)	3 (16.67)	13 (8.90)
Intellectual disability	6 (5.50)	2 (11.11)	2 (11.11)	10 (6.80)
Secondary diagnosis (*n*, %)				
Substance use	32 (29.36)	--	--	32 (21.90)
Personality disorder	27 (24.77)	4 (22.22)	2 (11.11)	33 (22.60)
Intellectual disability	3 (2.75)	7 (38.89)	3 (16.67)	13 (8.90)
Other	16 (14.68)	--	2 (11.11)	18 (12.30)
None noted	31 (28.44)	7 (38.89)	11 (61.11)	49 (33.60)

IQR, interquartile range

a. Descriptive information was unavailable for one patient.

### QPR

The QPR^11^ is a brief, self-report, personal recovery process measure designed based on qualitative interviews from people with psychosis.^12^ In its original form, the QPR comprised 22 items belonging to two subscales covering intrapersonal (e.g. personal autonomy, 17 items) and interpersonal factors (e.g. positive relationships, 5 items) known to facilitate recovery in people with psychosis. Each QPR item is a declarative statement with a 5-point Likert scale prompting the respondent to indicate how much they agree or disagree with the statement (scored 0–4). All items sum to a QPR total, with higher scores more indicative of recovery. The development study^11^ found good psychometric properties for the QPR in a self-selected sample of service users with experiences of psychosis (*N* = 111). Internal consistency (*α*) and 2-week test–retest reliability (*r*) were excellent for the larger Intrapersonal subscale (17 items, *α* = 0.94, *r* = 0.87), and acceptable for the smaller Interpersonal subscale (5 items, *α* = 0.77, *r* = 0.77). The QPR’s convergent validity was supported by correlational analyses with referential self-report measures of constructs related to recovery – personal empowerment, quality of life – and general psychiatric assessment tools such as the General Health Questionnaire. A subsequent study developed a revised short-form QPR with 15 items.^14^ In this study the following indices were used: QPR 22 Total (22 items, range 0–88), Intrapersonal (17 items, range 0–68), Interpersonal (5 items, range 0–20) and QPR 15 Total (15 items, range 0–60). To the authors’ knowledge, there are no published psychometric evaluations of the QPR in forensic populations.

### Statistical analysis

Data were merged and cleaned. Data analysis was undertaken in R version 4.4.2 for Windows^22^ using the packages psych,^23^ FSA^24^ and lavaan.^25^ Score indices were computed from the raw item data, then checked for normality by examining skew and kurtosis values and using the Shapiro–Wilk test. These tests indicated moderate violations to the assumption of normality. The decision was therefore made to use non-parametric tests where possible in subsequent analyses, and median and interquartile range are reported for measures of central tendency and spread, respectively.

The test structure of QPR was first evaluated using confirmatory factor analysis (CFA), with fit assessed for the original two-factor structure.^11^ Because CFA showed poor fit, exploratory factor analysis (EFA) was undertaken on the covariance matrix. Principal axis factoring (PAF) and oblimin rotation were used to identify the latent factors in EFA. PAF is robust to mild deviations from normality, as with the present data.^26^ Oblimin rotation allows for correlation of resulting factors should this be supported by the underlying data. Unique QPR score indices were produced following factor analysis; factor scores were derived by summing the raw item scores corresponding to all items loading on the resulting factors. The association between participant characteristics and these factor scores was explored using the tests of association described below.

The criterion validity of the QPR was assessed by comparing QPR scores according to participants’ current setting. Spearman’s rho (*ρ*) was used to quantify the association between QPR score indices, and the Kruskal–Wallis test to compare groups. Kruskal–Wallis effect sizes are reported as eta^2^.^27^ Dunn’s test with the Holm correction for multiple comparisons was used to explore significant group differences. The participant variables explored included age, primary diagnosis type, current setting (groupings: high-, medium-/low-security or open wards and the community) and the length of time spent in their current setting. Internal consistency and test–retest reliability (in a subsample, *n* = 50) were assessed using the intraclass correlation coefficient (ICC) following methods described by Koo and Li.^28^ Specifically, for internal reliability the intraclass correlation coefficient was calculated using a two-way, mixed-effects model with an average measure and absolute agreement. For test–retest reliability, the ICC was calculated using a two-way, mixed-effects model, single measurement and absolute agreement.

## Results

### Factor analysis

The Kaiser–Meyer–Olkin Measure of Sampling Adequacy^29^ was 0.90, and for each of the QPR items was >0.70, indicating that sample size was adequate for factor analysis.^30^ Bartlett’s Test of Sphericity was highly significant (*χ* ^2^(231) = 1675.784, *P* < 0.001), indicating that correlation between items was sufficiently large for factor analysis.^30^

A CFA was conducted to test fit to the original two-factor structure.^11^ Robust maximum likelihood estimation was used, given moderate violations of normality. The CFA demonstrated poor overall fit (*χ* ^2^(208) = 376.5, *P* < 0.001), and both incremental and absolute fit indices fell below recommended cut-offs for acceptable fit (robust comparative fit index 0.84, robust Tucker–Lewis index 0.82, robust root mean square error of approximation 0.09, standardised root mean squared residual 0.07).^31^ Factor analysis was then undertaken to explore the factor structure; parallel analysis indicated that two factors should be retained.^32^ All 22 QPR items were submitted for exploratory factor analysis using PAF and oblimin rotation on the covariance matrix, facilitating comparison with the original QPR factor analysis.^11^ Although the initial model accounted for 45% of total variance, 7 items did not have a salient primary factor loading of >0.4 and were therefore not contributing to the underlying factor structure.^33^ Starting with items with the lowest primary loading, those without a salient primary loading were removed and the model respecified, in iteration, until all remaining items loaded >0.4. Thereafter, items with high cross-loadings (>0.3) would be dropped.^34^ Item communalities were examined, with consideration being given towards removal for items with communalities <0.4.^34^ No items were removed based solely on cross-loadings or low communalities. The final model contained 16 items and accounted for 36% of variance (23% Factor 1, 13% Factor 2). QPR items 3, 5, 6, 9, 10 and 16 failed to contribute to the 2-factor structure and were excluded from the final model. The unstandardised, rotated loadings from the pattern matrix in the final model are presented in Table 2. Internal reliability of the two factors was good (Table 3).

**Table 2:** Pattern matrix resulting from exploratory factor analysis of the Questionnaire about the Process of Recovery (*n* = 145)^a^

Item	Factor analysis	Factor	
		1	2
	Percentage variance accounted for	23	13
	Cumulative percentage variance accounted for	23	36
	Item	Loading	
22	‘I can find the time to do the things I enjoy’.	0.78	
12	‘I can take charge of my life’.	0.76	
4	‘I feel part of society rather than isolated’.	0.75	
2	‘I feel able to take chances in life’.	0.67	
19	‘I can actively engage with life’.	0.61	
1	‘I feel better about myself’.	0.52	
21	‘I can take control of aspects of my life’.	0.51	
17^b^	‘My recovery has helped challenge other peoples’ views about getting better’.	0.46	
13	‘I am able to access independent support’.	0.45	
18	‘I am able to make sense of my distressing experiences’.		0.70
8	‘I have been able to come to terms with things that have happened to me in the past and move on with my life’.		0.65
15^b^	‘I feel my experiences have made me more sensitive towards others’.		0.59
11	‘I am able to understand myself better’.		0.51
14^b^	‘I can weigh up the pros and cons of psychiatric treatment’.		0.44
7	‘My experiences have changed me for the better’.		0.42
20^b^	‘I realise that the views of some mental health professionals is not the only way of looking at things’.		0.40
3	‘I am able to develop positive relationships with other people’.		
5	‘I am able to assert myself’.		
6	‘I feel that my life has a purpose’.		
9	‘I am basically strongly motivated to get better’.		
10	‘I can recognise the positive things I have done’.		
16^b^	‘Meeting people who have had similar experiences makes me feel better’.		

Loadings <0.30 are suppressed.

a. Permission to reproduce the QPR 22 items in this table was provided to the authors by Dr Sandra Neil.

b. Item is on the Interpersonal subscale reported by Neil et al.^11^

**Table 3:** Internal reliability and test–retest reliability coefficients and estimates

Questionnaire about the Process of Recovery (QPR) scale	*n*	Item	Internal reliability		Two-week test–retest reliability	
			ICC estimate (95% CI)	Rating^28^	ICC estimate (95% CI) (*n* = 50)	Rating^28^
QPR 22 Total	145	22	0.93 (0.91–0.95)	Excellent	0.75 (0.60–0.85)	Moderate to good
QPR Intrapersonal	145	17	0.93 (0.91–0.95)	Excellent	0.79 (0.65–0.87)	Moderate to good
QPR Interpersonal	146	5	0.65 (0.55–0.73)	Moderate	0.50 (0.26–0.68)	Poor to moderate
QPR Forensic Factor 1	145	9	0.89 (0.87–0.92)	Good to excellent	0.82 (0.71–0.90)	Moderate to excellent
QPR Forensic Factor 2	145	7	0.83 (0.79–0.87)	Good	0.63 (0.43–0.77)	Poor to good
QPR 15 Total	145	15	0.92 (0.90–0.94)	Excellent	0.81 (0.69–0.89)	Moderate to good

ICC, intraclass correlation coefficient.

The items on Factor 1 appear to be closely related to self-actualisation and empowerment, including those relating to autonomy and living a meaningful life. The items on Factor 2 appear to relate to growth and insight, involving reflection and self-examination, and refer to an increase in self-understanding and acceptance, perhaps resulting from such reflection and self-examination.

### Test scores and reliability

Item-level descriptive statistics and reliability are reported in Supplementary Table 1. There was evidence for moderate ceiling effects on a majority of the QPR 22 items and no evidence of floor effects, using the McHorney and Torlav^35^ threshold of 15% of the sample selecting the highest/lowest response options, respectively. Items with low item−total correlations were generally those on Forensic Factor 2, further supporting the measurement of a smaller, distinct latent construct within the QPR.

The ICC estimates for internal and test–retest reliability are reported in Table 3. Internal reliability ranged from ‘moderate’ to ‘excellent’ on all QPR score indices, with scales comprising few items, including QPR Interpersonal, ‘moderate’. Two-week test–retest reliability for Intra- and Interpersonal subscales was lower than that reported by Neil et al,^11^ with the latter subscale demonstrating ‘poor’ to ‘moderate’ test–retest reliability. Strong to very strong positive correlation coefficients were observed between all QPR score indices (all *ρ* > 0.65), presented in Table 4.

**Table 4:** Correlation matrix for Questionnaire about the Process of Recovery (QPR) score indices in the sample (*N* = 146)

QPR scale	QPR 22	QPR 22 Intrapersonal	QPR 22 Interpersonal	QPR Forensic Factor 1	QPR Forensic Factor 2
QPR 22 Intrapersonal	0.98^***^ (0.96–0.99)				
QPR 22 Interpersonal	0.78^***^ (0.69–0.83)	0.67^***^ (0.55–0.76)			
QPR Forensic Factor 1	0.95^***^ (0.91–0.97)	0.95^***^ (0.92–0.97)	0.69^***^ (0.58–0.77)		
QPR Forensic Factor 2	0.86^***^ (0.79–0.91)	0.84^***^ (0.76–0.90)	0.73^***^ (0.61–0.81)	0.73^***^ (0.63–0.82)	
QPR 15	0.97^***^ (0.94–0.98)	0.99^***^ (0.98–0.996)	0.65^***^ (0.52–0.73)	0.96^***^ (0.93–0.97)	0.82^***^ (0.74–0.88)

*** *P* < .001.

### Association with the forensic recovery journey

**Fig. 1:**
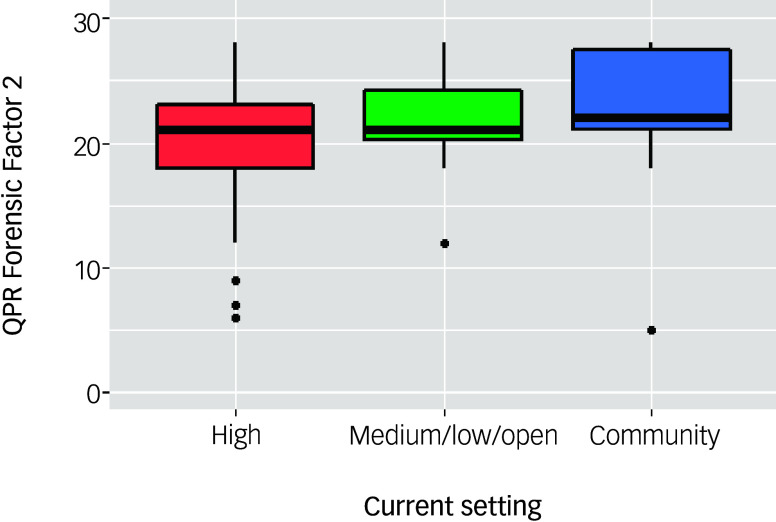
Boxplot presenting scores for Questionnaire about the Process of Recovery (QPR) Forensic Factor 2 by individuals’ current setting (*N* = 146). High, high-security; medium/low, medium-/low-security.

There were significant differences across current setting for participants’ self-rated recovery on all QPR score indices, except for the Interpersonal factor (Table 5). Effect sizes were small. Examining the descriptive statistics, scores on QPR 22 Total, QPR Intrapersonal, QPR Forensic Factors 1 and 2 and QPR 15 Total tended to increase as participants’ security level decreased. However, with the exception of QPR Forensic Factor 2, these significant *post hoc* comparisons did not survive Holm correction for multiple comparisons. Scores on QPR Forensic Factor 2, referred to as measuring growth and insight, were higher for those former high-security patients who were now in the community, compared with participants who were currently in a high-security setting (*Z* = −2.47, *P* = 0.04; Fig. 1).

**Table 5:** Scores for QPR indices (median (interquartile range)) across the forensic sample and by current setting

Questionnaire about the Process of Recovery (QPR) scale	Whole sample (*N* = 146)	Current setting			Kruskal–Wallis test statistic
		High (*n* = 109)	Medium-/low-security/open (*n* = 18)	Community (*n* = 18)	
QPR 22 Total	64.00 (9.50)	63.00 (13.00)	66.00 (14.80)	68.50 (19.00)	*χ* ^2^(2) = 6.57, *P* = 0.04, eta^2^ = 0.032
QPR Intrapersonal	50.00 (6.00)	50.00 (11.00)	51.00 (11.50)	52.50 (13.80)	*χ* ^2^(2) = 7.11, *P* = 0.03, eta^2^ = 0.036
QPR Interpersonal	15.00 (2.00)	14.50 (2.00)	15.00 (1.75)	15.00 (4.00)	*χ* ^2^(2) = 3.46, *P* = 0.18, eta^2^ = 0.01
QPR Forensic Factor 1	44.00 (6.50)	26.00 (6.00)	27.00 (6.50)	28.00 (9.50)	*χ* ^2^(2) = 7.67, *P* = 0.02, eta^2^ = 0.04
QPR Forensic Factor 2	26.00 (5.00)	21.00 (5.00)	21.00 (4.00)	22.00 (6.50)	*χ* ^2^(2) = 6.79, *P* = 0.03, eta^2^ = 0.034
QPR 15 Total	21.00 (4.00)	44.00 (9.00)	45.00 (11.00)	47.00 (13.00)	*χ* ^2^(2) = 7.50, *P* = 0.02, eta^2^ = 0.038

Table 6 presents the correlations between QPR score indices and individuals’ age and length of time they had spent in their current setting. Age was not related to any QPR score index (*P* > 0.05). Positive correlations were observed between some QPR score indices and the length of time that individuals had spent in their current setting (*ρ* coefficients 0.17−0.26).

**Table 6:** Correlation coefficients (Spearman’s *ρ*) and 95% confidence intervals for associations between QPR score indices and patient age and length of stay in current setting (*n* = 145)

Demographic factor	QPR 22	QPR 22 Intrapersonal	QPR 22 Interpersonal	QPR Forensic Factor 1	QPR Forensic Factor 2	QPR 15	Age
Age	0.07 (−0.10 to 0.24)	0.09 (−0.07 to 0.26)	−0.009 (−0.19 to 0.16)	0.07 (−0.10 to 0.24)	0.06 (−0.10 to 0.23)	0.10 (−0.07 to 0.27)	
Length of stay	0.24** (0.07 to 0.39)	0.25** (0.08 to 0.40)	0.17^*^ (0.0005 to 0.33)	0.25^*^ (0.07 to 0.41)	0.20^*^ (0.04 to 0.35)	0.26** (0.10 to 0.41)	0.42*** (0.28 to 0.55)

QPR, Questionnaire about the Process of Recovery.

* *P* < 0.05, **P < 0.01, ***P < 0.001.

## Discussion

This study aimed to explore differences in self-rated recovery among current and former forensic mental health service patients who had accessed high-security care. The validity of both the original 22-item^11^ and revised 15-item QPR^14^ was examined, alongside evaluation of the QPR test structure when applied within the forensic context.

On examination of test structure within the forensic context, the original structure of both intra- and interpersonal factors showed poor fit. Through EFA, a 2-factor model comprising 16 of the original 22 items was identified. Factor 1 was comprised of items that could be conceptualised as being supportive of self-actualisation and empowerment, with Factor 2 representative of growth and insight. Items 3, 5, 6, 9, 10 and 16 failed to load and were excluded from the final model. The internal reliability of Factor 1 was good to excellent and Factor 2 was good. While these are acceptable ratings, they contrast with QPR 22 Total, QPR Intrapersonal Total and QPR 15 total, which had excellent internal reliability. A similar picture was observed in terms of test–retest reliability with the original QPR scales, except for the QPR Interpersonal scale, rated at moderate to good. Forensic Factor 1 performed slightly better than the original QPR scales, at moderate to excellent, with Forensic Factor 2 displaying poor to good test–retest reliability. There are several possible explanations for Forensic Factor 2’s poorer test–retest reliability, particularly given this scale’s demonstrated ability to discriminate by recovery stage. First, this scale demonstrated lower internal consistency, which sets the upper limit for test–retest reliability, and it has fewer items, which reduces reliability. More conceptually, however, Forensic Factor 2 appears related to insight into one’s mental state, a characteristic known to fluctuate in people with psychosis.^36^ Additionally, the wording of some items may have been ambiguous (e.g. ‘I realise that the views of some mental health professionals is not the only way of looking at things’). Research shows these item characteristics introduce measurement unreliability^37^ and may account for the reduced test–retest reliability of this scale. Correlation coefficients among all indices, including the forensic factors, were *P* > 0.65, indicating strong positive relationships. Overall, this indicates that the two identified forensic factors have acceptable internal and test–retest reliability, although they performed slightly less well than the QPR 22- and 15-item totals.

When self-rated recovery was examined across secure settings and the community using all QPR indices and the newly identified forensic factors, all QPR score indices, except for QPR Interpersonal, produced significant results. Only the differences by setting in the new Forensic Factor 2 scores were sufficiently large to survive correction for multiple comparisons. In general, self-rated recovery increased as individuals progressed from high-secure through medium- and low-secure units to the community. Forensic Factor 2 items have been interpreted as representing growth and insight, essential requirements for complying with medication and other treatments to manage symptoms and reduce risk to self and others, both of which are requirements for acceptance to lower levels of security, or for transition to the community.^38^ Length of time at current location was also positively correlated with all QPR score indices, including both new forensic factors, indicating that self-rated recovery does increase with time spent in services appropriate to the individual’s needs. That Forensic Factor 2 discriminates based on current security setting aligns with findings reported by Rees and Thomson.^39^ In that 20-year follow-up study, psychiatric symptoms improved since participants had first been assessed in high-security settings, and qualitative interviews indicated that improved insight was pivotal to their personal recovery progress. While these correlations do not prove causation, it is encouraging that personal recovery increases as individuals progress through the forensic system.

The QPR is constructed with reference to the CHIME conceptual framework of recovery^40^ developed through systematic review and narrative synthesis of recovery experiences. CHIME refers to the identified recovery processes – connectedness, hope and optimism, identity, meaning and purpose, and empowerment. That QPR maps to the CHIME framework ensures that it measures recovery rather than elements of good practice within mental health services that foster recovery.^9^ While the QPR and the CHIME framework were developed from the experience of general adult mental health service patients with psychosis, Senneseth et al^41^ have mapped and extended the CHIME framework through thematic synthesis of personal recovery studies within forensic populations. They demonstrated that the core CHIME recovery processes remained relevant to individuals recovering within forensic mental health services, albeit these required to be played out within a context of restrictive practices and treatment requirements. An extension to the CHIME framework was made to capture self-management of risk as an additional recovery process relevant to those located with forensic mental health services. Central to this ‘safety and security’ recovery process was the concept of taking responsibility for remaining well and ownership of offending behaviour.^41^ It is suggested that, to enhance the validity of the QPR for use with forensic patients and ensure that the measure captures all forensic recovery processes, a small number of additional, declarative statements examining CHIME-Secure (CHIME-S) safety and security recovery process could be added, and the creation of a ‘QPR-forensic’ measure psychometrically explored.

Although the QPR assesses recovery, there are no available data to indicate that higher QPR recovery scores are associated with specific recovery outcomes, such as fewer readmissions or greater functional recovery. However, an association between higher self-rated quality of life among forensic patients was associated with lower scores on dynamic violence risk, and better recovery and functioning scores.^42^ Penney et al^43^ have also proposed an integrated conceptual model for use within the forensic environment that assimilates the components of risk, resilience and recovery, demonstrating the importance of personal recovery among forensic patients. Similarities in median scores across security-level groupings potentially suggest that individuals are able to find meaning and purpose in life regardless of their local environment.

While all QPR score indices, including the forensic factors, functioned well within this forensic population, there may be supporting evidence for embracing the use of the 16-item, 2-forensic factor version to describe forensic patients’ QPR scores. Through a mixed-methods validation of the 15-item QPR within a general rehabilitative mental health unit,^44^ qualitative findings examining content validity raised concerns that the 15-item QPR failed to capture the importance of interpersonal relationships for recovery. The 5-item interpersonal scale was removed from the original QPR 22-item measure to produce a slightly more psychometrically robust and less clinically burdensome measure.^8^ Within forensic services there is an emphasis on maintaining and rebuilding lost family and friend relationships, with the importance of external support to the recovery process being well recognised.^45^ Of the five QPR interpersonal scale items, in this sample one item loaded to Forensic Factor 1, three to Factor 2 and one failed to contribute. This loading pattern recognises the importance of interpersonal relationships to recovery in this population. Of note is that 5 items from the QPR 15 (items 3, 5, 6, 9 and 10) also failed to load to the forensic factors, further evidencing the nuanced nature of recovery within forensic settings. In considering the potential clinical burden upon individuals with severe mental illness, the 15-item QPR measure was regarded as being about the correct length.^44^

### Limitations

The majority of participants in this sample were located within a high-secure hospital. People within high-secure care have committed serious offences or display aggressive or violent behaviour, and this may impact upon their sense of self and, subsequently, the way in which they respond to QPR items. It may be useful to attempt to replicate this factor structure in a larger sample of forensic patients in medium- and low-secure settings and the community. At the time of study, high-secure services in Scotland admitted only men, while both men and women could be admitted to medium- and low-secure services. The cohort was created by recruiting current high-security patients and following previous high-security patients through services. This may have led to a gender bias that should be addressed through additional research. Three groups of data, collected at different time periods, were combined to create a larger sample size. The time difference could also include subtle changes in service delivery or the hospital environment, leading to bias.

In conclusion, this psychometric analysis found that the QPR is appropriate for use in assessing recovery among individuals accessing forensic mental health services. We provide evidence that a new 2-factor model can describe forensic patients’ recovery scores, using 16 of the original 22 items. This allows scores to be represented in a way that is more relevant for the recovery process along the forensic journey; factor analysis found latent factors related to self-actualisation and empowerment, as well as to growth and insight. While the core processes of recovery are considered to be the same whether a person is located in general adult or forensic mental health services, the context of restriction and compulsory treatment impacts upon those processes.^41^ While additional exploration with larger cohorts and examination of change over time are required to confirm factor structure within the original 22 items, QPR as used in a forensic sample may benefit from expansion to reflect the safety and security aspect of the CHIME-S framework for recovery within forensic mental health services. It should be considered whether additional declarative statements could be added to a forensic version of QPR, and be subject to subsequent psychometric evaluation. That 4 of the 5 omitted QPR interpersonal scale items loaded to the forensic factors, and 5 of the QPR 15 items did not contribute to the factor structure, lend weight to the need for a tailored measure of self-reported recovery for forensic mental health patients, which can be repeated to track progress in recovery as patients move through services.

## Supporting information

Gilling et al. supplementary material Gilling et al. supplementary material

## Data Availability

The anonymised dataset is available for request from the UK Data Service.

## Supplementary material

The supplementary material is available online at https://doi.org/10.1192/bjo.2025.10941
