# Supplementary material for: Measuring recovery in high-security patients: psychometric evaluation of the Questionnaire about the Process of Recovery and its utility to assess the forensic recovery journey
Source: BJPsych Open. 2026 Jan 22;12(1):e47. doi: 10.1192/bjo.2025.10941 (PMC12835695; doi:10.1192/bjo.2025.10941)
Supplement: Gilling et al. supplementary material [file S2056472425109411sup001.docx]

Method

*Dataset*

Anonymised QPR data and limited patient information originally collected in three studies related to forensic recovery were collated and analysed in the current study (N = 146). These studies included, 1. a 20-year follow up study exploring recovery outcomes of patients accessing high- security care in Scotland (1)(Thomson & Rees, 2023), 2. a research study (‘Recovery Research into Action’) which aimed in part to evaluate recovery measures in a high security sample (Health Research Authority IRAS ID 279735), and 3. a service evaluation of a psychological intervention delivered in high security, where the QPR was a primary outcome measure (2)(Gilling McIntosh, 2021). QPRs from the first administration in the service evaluation were collated for the present analyses.

*Questionnaire administration*

For two of the three studies (studies 1 and 2) contributing data to this current piece of work the QPR was delivered by a researcher, independent of the participant’s current or former clinical team to remove any bias, using a showcard detailing response choice. This approach was adopted primarily to ameliorate literacy issues, which can be compounded by psychosis, prevalent among forensic populations (3)(Svensson, 2015) and ensure attentional engagement. The process for delivering the QPR within the course of routine clinical care (study 3) was that a member of the psychology department provided varying levels of support as required for each individual to complete the QPR as part of a wider assessment battery prior to commencing a psychological intervention.

While it is recognised that utilising a mix of delivery methods has the potential to introduce biasing effects, evidence suggests that tool reliability remains high when delivered in alternative modes (4)(Cella et al 2015) although the literature is inconsistent regarding responses effects. Some studies indicate self-report wellbeing measures as providing more favourable results (5)(Anderson et al 1986), with others finding the opposite (6)(Chan et al 2004) or no significant differences based on mode of delivery (7)(Wu et al 1997). The QPR was also developed and initial psychometric assessment conducted using administered and self-complete modes (8) Neil et al 2009). That a mix of delivery methods was necessitated is therefore not considered influential to the findings.

Results

Table S1. Item-level descriptive statics and reliability (N = 146)

| QPR Item | Mean | Standard deviation | Skew | Kurtosis | Item- total correlation | Alpha if item dropped |
| --- | --- | --- | --- | --- | --- | --- |
| 1 | 2.95 | 0.90 | -1.02 | 1.21 | .74 | .92 |
| 2 | 2.51 | 1.01 | -0.87 | 0.08 | .63 | .93 |
| 3 | 2.95 | 0.82 | -1.02 | 1.63 | .62 | .93 |
| 4 | 2.30 | 1.15 | -0.39 | -0.91 | .63 | .93 |
| 5 | 2.72 | 0.94 | -1.22 | 1.12 | .61 | .93 |
| 6 | 2.93 | 0.85 | -1.03 | 1.40 | .67 | .93 |
| 7 | 2.98 | 0.96 | -1.08 | 0.92 | .67 | .93 |
| 8 | 2.83 | 1.05 | -0.98 | 0.32 | .70 | .93 |
| 9 | 3.24 | 0.72 | -1.16 | 2.82 | .62 | .93 |
| 10 | 3.01 | 0.77 | -1.28 | 2.97 | .73 | .93 |
| 11 | 2.98 | 0.77 | -0.97 | 1.70 | .73 | .93 |
| 12 | 2.82 | 0.96 | -0.88 | 0.39 | .70 | .93 |
| 13 | 3.00 | 0.75 | -1.06 | 2.06 | .66 | .93 |
| 14 | 2.95 | 0.87 | -1.43 | 2.68 | .49 | .93 |
| 15 | 2.88 | 0.92 | -1.15 | 1.44 | .45 | .93 |
| 16 | 2.86 | 0.87 | -0.74 | 0.34 | .51 | .93 |
| 17 | 2.82 | 0.86 | -0.76 | 0.71 | .63 | .93 |
| 18 | 2.82 | 0.91 | -0.99 | 0.87 | .64 | .93 |
| 19 | 2.94 | 0.90 | -1.16 | 1.70 | .78 | .92 |
| 20 | 2.92 | 0.79 | -0.87 | 1.20 | .44 | .93 |
| 21 | 3.03 | 0.78 | -1.18 | 2.60 | .73 | .93 |
| 22 | 2.86 | 0.99 | -1.07 | 0.69 | .67 | .93 |

References (supplemental material)

1. Thomson L, Rees C. Long-term outcomes of the recovery approach in a high-security mental health setting: a 20 year follow-up study. Frontiers in Psychiatry. 2023;14.

2. Gilling Mcintosh LM. An examination of the Forensic Matrix guide to delivering psychological therapies in forensic mental health services in Scotland. Edinburgh: University of Edinburgh; 2021.

3. Svensson I, Fälth L, Persson B. Reading level and the prevalence of a dyslexic profile among patients in a forensic psychiatric clinic. Journal of Forensic Psychiatry & Psychology. 2015;26(4):532-50.

4. Cella D, Hahn EA, Jensen SE, Butt Z, Nowinski CJ, Rothrock N, Lohr KN. Patient-Reported Outcomes in Performance Measurement. Research Triangle Park (NC): RTI Press; 2015.

5. Anderson JP, Bush JW, Berry CC. Classifying function for health outcome and quality-of-life evaluation. Self- versus interviewer modes. Med Care. 1986;24(5):454-69.

6. Chan KS, Orlando M, Ghosh-Dastidar B, Duan N, Sherbourne CD. The interview mode effect on the Center for Epidemiological Studies Depression (CES-D) scale: an item response theory analysis. Med Care. 2004;42(3):281-9.

7. Wu AW, Jacobson DL, Berzon RA, Revicki DA, van der Horst C, Fichtenbaum CJ, et al. The effect of mode of administration on medical outcomes study health ratings and EuroQol scores in AIDS. Qual Life Res. 1997;6(1):3-10.

8. Neil ST, Kilbride M, Pitt L, Nothard S, Welford M, Sellwood W, Morrison AP. The questionnaire about the process of recovery (QPR): A measurement tool developed in collaboration with service users. Psychosis. 2009;1(2):145-55.
